# Muscle-Specific Biomechanical Adaptations Following Rehabilitation Treatment in Cervical Spondylosis: A Pilot Study

**DOI:** 10.3390/life16010147

**Published:** 2026-01-16

**Authors:** Andreea Ancuța Talinga, Roxana Ramona Onofrei, Ada-Maria Codreanu, Veronica Aurelia Romanescu, Marius-Zoltan Rezumeș, Dan-Andrei Korodi, Oana Suciu, Claudia Borza

**Affiliations:** 1 Doctoral School, “Victor Babeş” University of Medicine and Pharmacy, Eftimie Murgu Sq. No. 2, 300041 Timişoara, Romania; andreea.vataman@umft.ro (A.A.T.); ada.codreanu@umft.ro (A.-M.C.); veronica.romanescu-pesecan@umft.ro (V.A.R.); marius.rezumes@umft.ro (M.-Z.R.); 2 Research Center for Assessment of Human Motion, Functionality and Disability, Department of Rehabilitation, Physical Medicine and Rheumatology, “Victor Babeş” University of Medicine and Pharmacy, Eftimie Murgu Sq. No. 2, 300041 Timişoara, Romania; oanasuciu78@umft.ro; 3 Center for Translational Research and Systems Medicine, Department of Functional Sciences–Pathophysiology, “Victor Babeş” University of Medicine and Pharmacy, Eftimie Murgu Sq. No. 2, 300041 Timişoara, Romania; borza.claudia@umft.ro; 4 Department of Medicine, Faculty of Medicine, “Vasile Goldiș” Western University of Arad, 310048 Arad, Romania; korodi.andrei@uvvg.ro

**Keywords:** cervical spondylosis, rehabilitation treatment, myotonometry, muscle stiffness, elasticity, dynamometry, biomechanical properties

## Abstract

Background. Cervical spondylosis is a degenerative disorder of the spine, frequently associated with chronic neck pain, reduced mobility, and functional impairment. Patients develop alterations in muscle tone, stiffness, and elasticity, which further contribute to disability. This study aimed to investigate the effects of a 14-day standardized rehabilitation program on the biomechanical and contractile properties of cervical and scapular muscles in patients with cervical spondylosis. Methods. This study used a single-group pre–post observational design on 23 patients (16 women, 7 men; mean age 61.1 ± 14.2 years) diagnosed with cervical spondylosis. All participants completed a standardized rehabilitation treatment that included cervical mobilization, stretching, isometric exercises, scapular stabilization, electrotherapy, ultrasound, thermotherapy, and balneotherapy. Muscle properties were evaluated bilaterally using the MyotonPRO^®^ device, measuring frequency, stiffness, decrement, relaxation time, and creep. Assessments were performed in a sitting position for the deltoid, upper trapezius and pectoralis major, both at baseline (T0) and after treatment (T1). Handgrip strength was assessed bilaterally with a handheld dynamometer. Results. The deltoid muscle showed a significant reduction in frequency (14.86 → 13.50 Hz, *p* = 0.034) and stiffness (306.4 → 256.1 N/m, *p* = 0.014) on the right side, suggesting normalization of tone and passive resistance. The upper trapezius had a significant bilateral decrease in decrement (*p* < 0.05), reflecting improved elasticity. The pectoralis major displayed the most consistent adaptations, with increased frequency (right side, *p* = 0.008), improved relaxation bilaterally (*p* < 0.05), and significant reductions in decrement and creep (*p* < 0.01). Handheld dynamometry confirmed increased handgrip strength, with a 5.4% improvement on the left side and 7.6% on the right side. Conclusions. In our study measurable changes in muscle parameters were observed following a rehabilitation program in patients with cervical spondylosis. The integration of myotonometry and dynamometry allowed objective assessment of muscle adaptations supporting the clinical value of individualized rehabilitation strategies.

## 1. Introduction

Cervical spondylosis is a degenerative disorder of the spine, with a significant impact on patients’ quality of life and functional capacity. It is a progressive degenerative process of the cervical intervertebral discs, associated with osteophytic changes in the vertebral bodies and arthropathy of the interapophyseal joints [1]. The degenerative process occurs physiologically with advancing age, but individual and occupational risk factors can accelerate its progression, leading to the early onset of clinical symptoms [2]. Cervical spondylosis affects over 85% of individuals over the age of 60 and is considered the main cause of chronic neck pain in the adult population [3]. At the same time, studies show that degenerative changes can begin as early as the third decade of life, with a variable degree of progression depending on genetic predisposition, biomechanics, and environmental factors [4].

However, the pathophysiological mechanisms involved in cervical spondylosis are complex and include progressive disc degeneration, dehydration of the nucleus pulposus, decreased intervertebral disc height, and the formation of marginal osteophytes. These changes cause segmental instability and compression of adjacent nerve and vascular structures, which explains the variability of the clinical picture [5]. Beyond local symptoms like neck pain, stiffness, neurological complications such as radiculopathy or cervical myelopathy are common reasons for seeking medical attention and can have severe functional consequences [6].

Recent study shows that patients with cervical spondylosis develop biomechanical changes in their muscles, with increased muscle tone, stiffness, and decreased tissue elasticity, which contributes to chronic pain and limited cervical mobility [3].

The selection of the upper trapezius, deltoid, and pectoralis major muscles for biomechanical assessment in this study was based on their major involvement in compensatory mechanisms associated with cervical spondylosis. The upper trapezius was assessed as being involved in cervical postural control and is often characterized by hypertonicity and increased stiffness in patients with chronic cervical pain and postural disorders like forward head posture [7]. In our study we also included the deltoid and pectoralis major muscles because they actively participate in shoulder girdle biomechanics and can reflect functional adaptations secondary to cervical dysfunction through muscle and postural chains [8]. In this study, we added myotonometry assessment because it provides useful information about muscle adaptations related to biomechanical changes in the head, neck, and shoulders [9]. Myotonometry is a reliable and accurate method for quantifying muscle biomechanical properties such as stiffness, tone and elasticity in various musculoskeletal conditions, including those affecting the cervical spine [10,11] and the assessment can reveal hyperactivity of the paravertebral muscles, functional asymmetry, and compensatory adaptations that can keep pain and dysfunction going in patients diagnosed with cervical spondylosis [12].

The main objective of the present study was to evaluate changes in the biomechanical parameters of the cervical and shoulder girdle muscles using myotonometry as well as handgrip strength assessed by a handheld dynamometer in patients diagnosed with cervical spondylosis, before and after a standardized 14-day rehabilitation treatment program.

## 2. Materials and Methods

This study used a single-group pre-post observational design, in which all participants received the same rehabilitation treatment. No control or comparison group was included. Our research was conducted in a medical rehabilitation clinic in Timis county and included 23 patients diagnosed with cervical spondylosis who were submitted for rehabilitation treatment between April to June 2025. The study was conducted in accordance with the Declaration of Helsinki and approved by the Ethics Committee of “Victor Babes” University of Medicine and Pharmacy Timisoara, Timisoara, Romania (protocol nr. 28/10.01.2022).

Inclusion criteria were confirmed diagnosis of cervical spondylosis (X-ray or magnetic resonance imaging), age between 27 and 79 years, presence of clinical symptoms such as neck pain and stiffness, and ability to attend daily rehabilitation sessions. The study included 23 patients, of whom 16 were women (69.6%) and 7 were men (30.4%). Among the female participants, only three women were under the age of 40 years (27, 35, and 39 years, respectively) and presented imaging-confirmed degenerative cervical changes. Exclusion criteria: history of cervical surgery, associated central or peripheral neurological diseases (e.g., multiple sclerosis, peripheral neuropathies), systemic inflammatory diseases (rheumatoid arthritis, spondyloarthritis), primary muscle disorders (myopathies, dystrophies), or inability to comply with the recovery protocol. All patients agreed to participate in the study and signed an informed consent.

### 2.1. Rehabilitation Treatment Protocol

The rehabilitation treatment protocol was complex as presented in Table 1. All interventions were applied in the same order for all participants.

The first procedure performed was balneotherapy, which consisted of applying CO_2_ mineral water baths at a temperature of 33 °C for 20 min per day. The baths were performed in rectangular acrylic tubs to maintain a constant water temperature throughout the procedure. After this procedure, the patients in the study had an hour of rest.

After the rest period, we continued with a session of Transcutaneous Electrical Nerve Stimulation (TENS), which is a low-frequency current therapy, with electrodes placed on the skin in the painful area in one and two circuits arranged paravertebrally in the cervical region, the intensity being felt as vibration with analgesic effects, 15 min duration. In this study, ultrasound therapy was also applied to patients, in the cervical paravertebral area, with an intensity of 0.5 W/cm^2^, 5 min per day, followed by a thermotherapy session consisting of “cape-style” wax wraps covering the cervical area and both shoulders, at 40–45 degrees for 20 min per session. After this procedure, they had one hour of rest. The rehabilitation treatment protocol was completed by a kinetotherapy session that included active and passive cervical mobilization exercises, paravertebral muscle stretching exercises, isometric and scapular stabilization exercises, each of them including 3 sets of 10 to 12 repetitions, with short breaks between sets. The duration was 40 min daily for 14 consecutive days.

### 2.2. Muscle Properties Assessment

Objective assessment of muscle properties was performed using the MyotonPRO^®^ device (Myoton AS, Tallin, Estonia), a validated non-invasive portable device for quantifying mechanical muscle characteristics [13].

MyotonPRO emits a short, low-intensity mechanical stimulation on the skin, causing tissue oscillation that is recorded by an acceleration sensor. Analysis of the oscillations allows specific biomechanical parameters to be calculated [11].

We analyzed the following parameters: frequency which is an indicator of muscle tone at rest, stiffness representing muscle resistance to external deformation, logarithmic decrement that measure tissue elasticity, relaxation time representing the time required for the muscle to return to its initial state after contraction and creep, which is the ability of tissue to gradually adapt to a sustained force [14].

**Figure 1:**
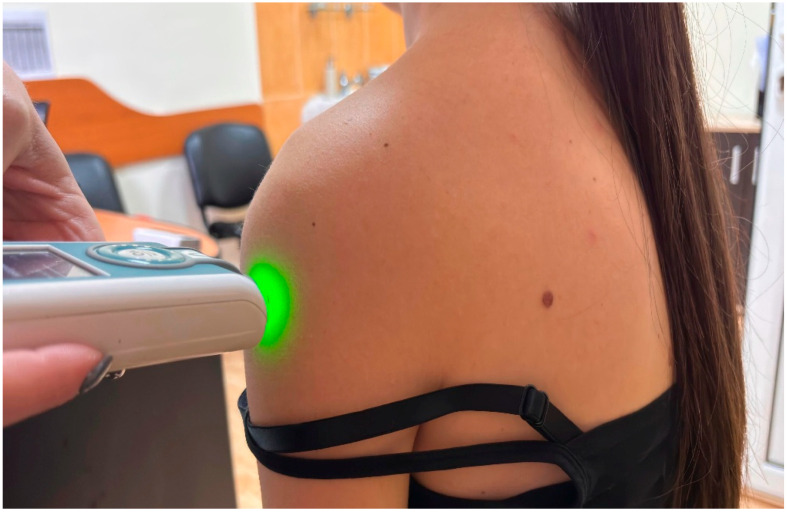
Example of myotonometric measurement of the deltoid muscle.

**Figure 2:**
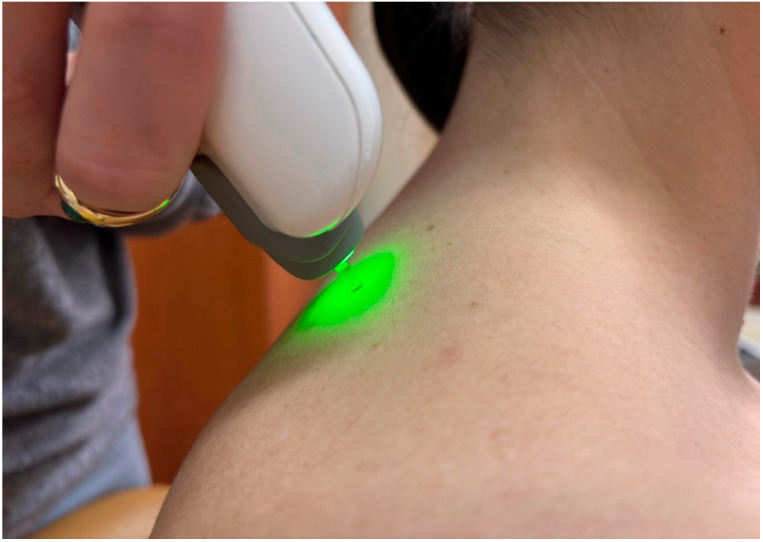
Example of myotonometric measurement of the trapezius muscle.

All myotonometric measurements were performed in the sitting position, with participants seated on a standardized chair, hips and knees flexed at approximately 90°, feet resting flat on the floor, and upper limbs in a neutral relaxed position alongside the body [15]. The sitting position was selected because it assures stability and reduces variability associated with gravitational effects in standing or supine positions. Measurement sites were identified by palpation using anatomical landmarks and labelled before data collection. The probe was placed perpendicular to the skin surface at the predetermined location during both the first and last assessments. For deltoid muscle, the probe of the MyotonPro was positioned at the mid-belly of the deltoid, approximately halfway between the lateral border of the acromion and the insertion on the deltoid tuberosity of the humerus (Figure 1), with the arm resting neutrally [16]. Upper trapezius was assessed at the midpoint between the spinous process of C7 and the lateral tip of the acromion, corresponding to the thickest portion of the muscle belly (Figure 2), and pectoralis major muscle was measured at the sternal portion, in the middle distance between the lateral border of the sternum and the humeral insertion, with the arm relaxed along the trunk [17].

Measurements were performed by the same examiner to reduce interobserver variability. Patients were assessed under standardized ambient temperature conditions and within a constant time window to limit circadian variations. For each muscle and pa-rameter, five successive measurements were taken, bilaterally, and the arithmetic mean was used in the analysis. The assessments were performed on day 0 (before the start of the rehabilitation treatment) and on day 14 (at the end of the rehabilitation treatment).

### 2.3. Muscle Strength Assessment Using Dynamometry

In our study, we used a handheld dynamometer (HHD)—MicroFET2 dynamometer (Hoggan Scientific, LLC, Salt Lake City, UT, USA), a device used to assess handgrip strength [18]. For the testing procedure, patients were familiarized with the device through a trial test, then handgrip strength was tested in proper positions for both the right and left upper limbs. Values were expressed in kilogram-force, subsequently converted to newtons; 1 kgf = 9.80665 N. For each patient, three measurements were taken, each lasting 5 s, with a 30-s break between repetitions. The average value of the three assessments was recorded. The measurements were taken bilaterally, both before treatment (day 0) and after treatment (day 14).

Statistical analysis was performed with MedCalc Statistical Software version 23.2.1 (MedCalc Software Ltd., Ostend, Belgium). Data in this study are presented as means and standard deviations. Shapiro–Wilk test was used to test normality. Paired *t*-tests were applied for normally distributed variables, while the Wilcoxon test was used for non-normally distributed data to compare measurements before and after rehabilitation treatment. In addition to statistical significance, effect sizes were calculated to quantify the observed changes. For paired *t*-tests, Cohen’s *d* was calculated as the mean difference between post- and pre-intervention values divided by the standard deviation of the paired differences (*d* =Mean_difference_/SD_difference_) [19]. For non-parametric comparisons, the effect size *r* was obtained from the Wilcoxon test as *r* = Z/√N, where N represents the number of paired observations [20,21]. A value of 0.8 is considered a large effect size, a value of 0.5 is considered a medium effect size, and a value of 0.2 is considered a small effect size. A significance level of 0.05 was set.

## 3. Results

The study group included 23 patients aged between 27 and 79 years, mean age 61.1 ± 14.2 years (69.6% women and 30.4% men) met the inclusion criteria and were included in the study. Patients’ characteristics are presented in Table 2.

Myotonometry parameters were assessed before rehabilitation treatment (T0) and 14 days after (T1) on both the right and left sides. All assessments for the deltoid muscle are shown in Table 3.

Myotonometric analysis of the deltoid revealed moderate but suggestive changes. Frequency showed a significant reduction on the right side in the sitting position (*p* = 0.034), while no significant differences were found on the left side (*p* = 0.596). Stiffness of the deltoid muscle measured on the right side decreased significantly from 306.4 N/m to 256.1 N/m, *p*-value was 0.0149, while for the left side, changes were not significant (*p* = 0.803). Relaxation, decrement, and creep parameters did not show significant changes (*p* > 0.05). Overall, the rehabilitation program showed a clear reduction in deltoid tone and stiffness, more pronounced on the right side, along with a tendency toward better elasticity and relaxation.

The upper trapezius muscle did not show significant changes in frequency and stiffness, but the decrement decreased significantly bilaterally (right *p* = 0.049; left *p* = 0.042), indicating an increase in muscle elasticity (Table 4). Relaxation in the left trapezius muscle had an increasing trend after rehabilitation treatment (*p* = 0.088), without reaching the limit of significance. The creep parameter did not show significant changes.

While the deltoid and trapezius muscles showed a reduction in tone and stiffness, the pectoralis major reacted differently (Table 5). The pectoralis major muscle showed the most consistent effects: significant increase in frequency on the right side (*p* = 0.0085), improvement in bilateral relaxation time (right *p* = 0.0045; left *p* = 0.0317), decrease in decrement (right *p* < 0.0001; left *p* = 0.0006) and reduction in creep (right *p* = 0.0045; left *p* = 0.0345). This biomechanical profile may suggest functional adaptation, characterized by increased muscle tone and elasticity, potentially related to the stabilizing role of the pectoralis major during rehabilitation.

The assessment of handgrip strength in the upper limbs revealed an overall increase after the 14-day rehabilitation program. Mean values indicated an improvement in grip strength on both the left and right sides (Table 6). More specifically, in the left upper limb, the mean handgrip strength increased from 332.93 N to 350.88 N, representing an improvement of approximately 5.4%. In the right upper limb, the increase was more evident, from 338.91 N to 364.51 N, corresponding to an improvement of 7.6%. As minimal clinically important differences for handgrip strength are not uniformly established across populations, the clinical relevance of these changes should be interpreted cautiously [22]. In our study, handgrip strength was used as a general measure of upper-limb neuromuscular performance, in line with previous methodological approaches [23].

## 4. Discussion

The results of this study present objective evidence of the beneficial effects of medical rehabilitation treatment on the biomechanical properties of cervical and scapular muscles in patients with cervical spondylosis. The best effects were observed in the deltoid muscle, where a significant reduction in frequency and stiffness was observed on the right side with an improvement in biomechanical parameters, namely relaxation and elasticity.

Our investigations are similar to those of Guduru et al., who reported a decrease in posterior deltoid stiffness after postural correction in subjects with rounded shoulders [9], as well as those of Kurashina et al., who demonstrated an increase in anterior deltoid stiffness in adhesive capsulitis and its reduction following therapeutic intervention [16]. These data indicate that deltoid stiffness is a sensitive biomarker of treatment response in cervical musculoskeletal disorders. In the upper trapezius the changes were smaller with insignificant reductions in frequency and stiffness, but with a statistically significant bilateral decrease, reflecting an improvement in tissue elasticity. This is relevant to our study because the trapezius is one of the muscles most associated with chronic neck pain and maladaptive postural loading [24].

A meta-analysis performed by Opara et al. confirmed higher values of stiffness in the upper trapezius in patients with chronic neck pain compared to healthy subjects in the control group [25]. In addition, Kim et al. demonstrated that cervical and scapular stabilization programs reduce trapezius stiffness and improve cervical mobility [26], while Wendt et al. highlighted the relationship between trapezius stiffness and limitations in cervical rotation [27]. The results of our study suggest that an improvement in the elasticity of the upper trapezius muscle after medical rehabilitation treatment helps restore cervical segment mobility and reduce functional disability.

A special aspect of our research was observed in the results obtained for the pectoralis major muscle. Unlike the deltoid and trapezius, the pectoralis major showed a significant increase in frequency and stiffness on the right side, accompanied by improvements in elasticity and bilateral relaxation capacity. This biomechanical profile reflects a functional adaptation to the load requirements of medical rehabilitation treatment. This interpretation is supported by Reiner et al., who demonstrated that static stretching alone does not alter the stiffness of the pectoralis major muscle, but improves contractile performance [28].

The results of our study suggest that the physical therapy exercises induce a mixed adaptation, namely higher tone combined with improved elasticity, as compared to the maladaptive stiffness described by Kim et al. [26]. It is important to mention that the improvements observed in myotonometric assessment parameters were accompanied by an increase in handgrip strength with more evident results on the right side. The close relationship between passive biomechanical markers such as tone, stiffness, elasticity, and hand grip strength reinforces the importance of using multimodal assessments in rehabilitation treatment.

From a clinical perspective, these results highlight the importance of targeted rehabilitation treatment strategies that address not only overall cervical mobility but also specific adaptations of individual muscle groups. While the deltoid and trapezius benefited from normalization of tone and increased elasticity, the pectoralis major muscle had a functional adaptation reflecting tissue stabilization. These muscle changes reflect the need to adjust medical rehabilitation treatment according to the biomechanical profile of each muscle involved in cervical spondylosis.

This study has several limitations. One limitation is the relatively small sample size, as this study is considered a pilot study. Moreover, analyses were performed on the entire sample without subgroup comparisons based on sex, age, or severity of cervical spondylosis, and this restricts the generalizability of the results. Another limitation is the fact that no formal correlation analysis was conducted between myotonometric parameters and handgrip strength; only parallel changes were reported. Future studies should include a larger number of patients with different grades of severity of cervical spondylosis, as well as a control group to improve the clinical relevance of the results.

Despite these limitations, the simultaneous use of myotonometry and dynamometry is a particular advantage of the study, allowing for an objective and detailed characterization of muscle adaptations.

## 5. Conclusions

In our study, significant changes in the biomechanical and contractile properties of the cervical and scapular muscles were observed in patients with cervical spondylosis following a rehabilitation treatment. In the absence of a control group, these findings should be interpreted with caution. The deltoid muscle showed a significant reduction in frequency and stiffness, especially on the right side, suggesting normalization of resting tone and decreased passive resistance. The upper trapezius showed a significant bilateral improvement in tissue elasticity, reflected in a reduction in decrement, and the pectoralis major muscle demonstrated a complex adaptive response, characterized by increased tone and stiffness on the right side accompanied by significant improvements in elasticity and relaxation capacity bilaterally.

The results highlight the role of objective assessment tools, such as myotonometry and dynamometry for monitoring muscle-related changes during rehabilitation treatment in patients with cervical spondylosis. Further controlled studies are required to clarify the role of the rehabilitation treatment in the observed changes.

## Figures and Tables

**Table 1:** Summary of the Rehabilitation Treatment Protocol.

Component	Description	Duration/Dosage	Frequency
Balneotherapy	CO_2_ mineral water bath, 33 °C	20 min/session	Daily, 14 days
TENS	Paravertebral cervical application, analgesic intensity	15 min/session	Daily, 14 days
Ultrasound therapy	Cervical region, 0.5 W/cm^2^	5 min/session	Daily, 14 days
Thermotherapy	Paraffin wax wraps (cape-style), 40–45 °C	20 min/session	Daily, 14 days
Kinetotherapy	Cervical mobilization, stretching, isometric and scapular stabilization exercises	3 sets × 10–12 reps per exercise	Daily, 14 days

TENS—transcutaneous electrical nerve stimulation; reps—repetitions; CO_2_—carbon dioxide. W/cm^2^, ultrasound intensity expressed as watts per square centimeter. Temperatures are expressed in degrees Celsius (°C).

**Table 2:** Patients’ characteristics.

	Patients (n = 23)
Age (years) mean ± SD	61.17 ± 14.19
Weight (kg) mean ± SD	73.95 ± 15.79
Height (cm) mean ± SD	164.17 ± 8.39
BMI (kg/m^2^)	27.33 ± 4.84

Data are presented as mean ± standard deviation. BMI—body mass index.

**Table 3:** Biomechanical parameters of the deltoid muscle tested using MyotonPRO.

Parameter	Right Side T0	Right Side T1	*p*-Value	Effect Size (Cohen’s d/r)	Left Side T0	Left Side T1	*p*-Value	Effect Size (Cohen’s d/r)
Frequency (Hz) (Mean ± SD)	14.86 ± 2.53	13.50 ± 1.27	0.034	0.47	13.98 ± 2.42	13.49 ± 2.73	0.596	0.11
Stiffness (N/m) (Median [IQR])	285.0 [263.8–317.0]	254.0 [235.5–277.7]	0.014	−0.51	280.3 ± 68.2	274.7 ± 66.5	0.803	−0.05
Relaxation (ms) (Mean ± SD)	19.08 ± 3.72	20.33 ± 4.18	0.357	0.2	20.34 ± 3.91	19.53 ± 4.71	0.579	−0.12
Creep (Mean ± SD)	1.21 ± 0.28	1.32 ± 0.30	0.254	0.24	1.29 ± 0.26	1.23 ± 0.30	0.568	−0.12
Decrement (Median [IQR])	1.39 [1.27–1.70]	1.32 [1.20–1.43]	0.532	−0.13	1.52 ± 0.29	1.44 ± 0.31	0.428	−0.17

Data are presented as mean ± standard deviation (SD) or median and [interquartile range, IQR]. T0 indicates baseline assessment, and T1 indicates post-treatment assessment. Hz, hertz; N/m, newtons per meter; ms, milliseconds. Effect size is reported as Cohen’s d or r, as appropriate.

**Table 4:** Biomechanical parameters of the Upper trapezius muscle tested using MyotonPRO.

Parameter	Right Side T0	Right Side T1	*p*-Value	Effect Size (Cohen’s d/r)	Left Side T0	Left Side T1	*p*-Value	Effect Size (Cohen’s d/r)
Frequency (Hz) (Mean ± SD)	18.55 ± 3.94	17.73 ± 3.14	0.456	0.16	18.19 ± 4.09	18.22 ± 3.21	0.977	0.01
Stiffness (N/m) (Mean ± SD)	402.6 ± 96.49	389.0 ± 67.09	0.605	0.11	392.4 ± 107.3	385.5 ± 81.36	0.792	0.06
Relaxation (ms) (Median [IQR])	13.3 [12.05–17.02]	13.5 [12.07–14.95]	0.584	0.11	15.5 ± 4.3	13.7 ± 3.14	0.088	0.37
Creep (Median [IQR])	0.87 [0.80–1.14]	0.89 [0.79–0.99]	0.637	0.10	0.99 ± 0.26	0.92 ± 0.20	0.285	0.23
Decrement (Mean ± SD)	1.57 ± 0.30	1.42 ± 0.24	0.049	0.43	1.57 ± 0.22	1.41 ± 0.25	0.042	0.45

Data are presented as mean ± standard deviation (SD) or median [interquartile range, IQR]. T0 indicates baseline assessment, and T1 indicates post-treatment assessment. Hz, hertz; N/m, newtons per meter; ms, milliseconds. Effect size is reported as Cohen’s d or r, as appropriate.

**Table 5:** Biomechanical parameters of the pectoralis major muscle tested using MyotonPRO.

Parameter	Right Side T0	Right Side T1	*p*-Value	Effect Size (Cohen’s d/r)	Left Side T0	Left Side T1	*p*-Value	Effect Size (Cohen’s d/r)
Frequency (Hz) (Median [IQR])	12.9 [11.65–17]	18.4 [13.77–20.97]	0.008	0.55	15.92 ± 3.43	17.56 ± 4.02	0.158	0.3
Stiffness (N/m) (Mean ± SD)	317.6 ± 80.53	369.9 ± 90.79	0.072	0.39	361.3 ± 99.19	387.8 ± 85.35	0.275	0.23
Relaxation (ms) (Mean ± SD)	18.2 ± 3.72	14.6 ± 3.32	0.004	0.66	16.03 ± 4.49	13.88 ± 2.96	0.031	0.48
Creep (Median [IQR])	1.17 [0.95–1.3]	0.85 [0.80–1.03]	0.004	0.59	0.99 [0.85–1.11]	0.86 [0.80–0.96]	0.034	0.44
Decrement (Mean ± SD)	1.77 ± 0.38	1.33 ± 0.21	<0.001	1.05	1.77 ± 0.34	1.42 ± 0.26	0.001	0.84

Data are presented as mean ± standard deviation (SD) or median [interquartile range, IQR]. T0 indicates baseline assessment, and T1 indicates post-treatment assessment. Hz, hertz; N/m, newtons per meter; ms, milliseconds. Effect size is reported as Cohen’s d or r, as appropriate.

**Table 6:** Handgrip strength measured before and after rehabilitation treatment.

Handgrip Strength	Mean ± SD (kg/f)	Mean ± SD (N)
Right Side T0	34.56 ± 10.21	338.91 ± 100.12
Right Side T1	37.17 ± 10.13	364.51 ± 99.34
Left side T0	33.95 ± 10.83	332.93 ± 106.2
Left side T1	35.78 ± 10.73	350.88 ± 105.22

Handgrip strength data are presented as mean ± standard deviation (SD). kgf denotes kilogram-force; values expressed in newtons (N) were obtained by conversion (1 kgf ≈ 9.81 N). T0 indicates baseline assessment, and T1 indicates post-treatment assessment.

## Data Availability

The data presented in this study are available on request from the corresponding author due to privacy.

## Abbreviations

The following abbreviations are used in this manuscript:

BMI	body mass index
CO_2_	carbon dioxide
HHD	hand-held dynamometer
Hz	hertz
IQR	interquartile range
kgf	kilogram-force
N	newton
SD	standard deviation
T0	baseline assessment
T1	post-treatment assessment
TENS	transcutaneous electrical nerve stimulation
